# Rodent-borne pathogens as economic and zoonotic health threat to livestock farming: a review

**DOI:** 10.1016/j.onehlt.2026.101448

**Published:** 2026-05-16

**Authors:** Florian Huels, Jens Jacob

**Affiliations:** a Julius Kühn-Institute (JKI), Federal Research Centre for Cultivated Plants, Institute for Epidemiology and Pathogen Diagnostics, Rodent Research, Muenster, Germany

**Keywords:** Economic losses, Farming, Health, Livestock, Pathogens, Rodents

## Abstract

**Introduction:**

Economic losses and health risks caused by disease outbreaks in farm animals pose a considerable threat worldwide. Rodents play a crucial role in the transmission of a variety of zoonotic pathogens acting as intermediate, main or reservoir hosts for many bacteria, viruses and parasites. Rodent-borne pathogen transmission can occur through direct or indirect contact, through contaminated food and water or through arthropod vectors. This review provides an overview about the current state of knowledge to demonstrate how rodents are involved in the transmission of the most relevant livestock pathogens.

**Material and methods:**

Two literature databases (Web of Science, PubMed) were used to create a list of publications on all relevant pathogen types (bacteria, viruses, parasites, other), the most mentioned rodent species involved in pathogen transmission and the most common livestock types (cattle, swine, poultry) published in English from inception to September 2025.

**Results:**

The 494 articles that matched keywords/abstract and focus considered bacteria (n=204) as the main pathogen type, followed by parasites (n=166), viruses (n=92), others (n=15) and rodents on farms (n=17). The most studied pathogens were *Leptospira*, *Salmonella*, *Toxoplasma*, and *Trichinella*. Most papers concentrated on commensal *Rattus* spp. and *Mus musculus* – wild rodent species are clearly underrepresented and there were mostly descriptive studies without consideration of processes and intervention.

**Conclusion:**

The role of rodents in transmission pathways of an extremely broad range of pathogens is demonstrated. Further research is needed to understand the mechanisms to decrease economical cost and health issues for commensal and field/forest rodent species alike.

## 1 Introduction

Over the past 50 years, the global meat production increased rapidly due to growing demand resulting from the sharp rise in human population size. According to the Organisation for Economic Co-operation and Development (OECD), the world produced more than 350 million tonnes of meat in 2024 [1]. Global most dominant livestock types are poultry, cattle, pigs, sheep and goats [2]. However, the distribution of meat types varies worldwide depending on the region. In meat-producing agriculture in particular, humans, livestock as well as rodents and other wildlife live in close proximity facilitating transmission of pathogens from rodents to livestock [3], [4], [5], [6], [7], [8] and to humans [5], [9], [10], [11]. Rodents are common on and around farms due to the availability of food and shelter [12]. Commensal rodents damage building structures, damage and contaminate stored produce and water [7], [13], [14], [15].

Commensal rodents are a reservoir for many zoonotic pathogens, however, they frequently also act as an epidemiological vector in the transmission pathway. Adaptations to various environmental conditions enable them to inhabit households and livestock facilities year round [16]. They can transfer pathogens within and among farms [17]. Various rodent species are mentioned as priority factors in pathogen transmission on farms, in Europe mainly roof rat (*Rattus rattus*) [17], [18], [19], brown rat (*Rattus norvegicus*) [20], [21], [22], [23], house mouse (*Mus musculus*) [24], [25], [26], [27] and bank vole (*Clethrionomys glareolus*) [26], [28], [29]. Many studies show that rodents are often infected with a wide range of different pathogens and parasites such as *Leptospira* spp. [30], [31], [32]*, Toxoplasma gondii* [33], [34], [35], [36], *Coxiella burnetii* [37], [38], *Salmonella* spp. [39], [40], [41], [42] and *Trichinella* spp. [43], [44]. Many other pathogens associated with rodents play an important role in terms of economic losses and public health. Many of these pathogens can pose a risk to humans and therefore, some human infections/diseases are also considered. The ‘One Health’ approach represents a holistic strategy for preventing risks to animal and human health. From this perspective, livestock health and human health are directly linked. In the context of One Health, livestock farming includes not only farm animals but also people associated with the farm environment such as farm workers, a highly relevant risk group. However, the main objective of this review is to examine the role of rodents in pathogen transmission to livestock, to reflect the current state of scientific knowledge on this topic and to identify knowledge gaps.

## 2 Material and Methods

### 2.1 Literature search

This review considered all relevant papers that had been published until 30^th^ September 2025. Web of Science and PubMed were searched for publications in English language from inception of the journal. The search in title and abstracts comprised three groups of terms using Boolean operators ‘AND/OR’ and placeholder ‘*’: ‘rodent*’ (1) AND, ‘diseas* OR pathogen* OR parasit*’ (2) AND ‘farm animal* OR livestock* OR pig* OR poultr* OR broiler* OR layer hen* OR cattle*’ (3). Database entries containing at least one search term connected to one of the other groups were included in the review. The literature search comprised the three main steps identification, screening and final inclusion of publications. The identification and screening steps yielded increasingly finer specific exclusion procedures to gain a final list with relevant, topic-related publications.

### 2.2 Inclusion criteria

The articles had to describe the role of rodents in transmission of bacteria, viruses and parasites to livestock. The search mainly yielded epidemiological as well as experimental studies describing certain rodent species transmitting certain pathogens to specific livestock species. Furthermore, publications that focussed on certain livestock (cattle, poultry, pigs) and specific pathogens but not on rodents specifically were also included, when it was mentioned that rodents can play a decisive role in the transmission of these pathogens.

### 2.3 Exclusion criteria

The first exclusion step rejected irrelevant records and records which occurred in both Web of Science and PubMed. Articles focussing on rodents without reference to pathogen transmission or farm animal hazards were excluded. Publications that dealt with the epidemiology of the relevant diseases and pathogens as well as studies on agricultural science relating to livestock farming were removed. In a second more thorough analysis, further irrelevant publications were removed from the collection. Certain target species (livestock, humans) and several publications that deal primarily with farm hygiene and rodent management without connection to pathogen transmission from rodents to livestock were excluded.

The potential bias due to the concentration on articles in English is mitigated because there are 366 articles (74%) in English covering regions where other languages prevail. Including publications in any other language or “grey literature” may have further lessened bias but not removed it completely. This is a common issue in reviews [45], [46].

### 2.4 Pooling of prevalence data

Data from the included publications were pooled to synthesise prevalence estimates for pathogens and parasites in both livestock and small mammals. Prevalence or seroprevalence data were compiled from all relevant field studies that identified at least one specific pathogen or parasite in one of the major livestock species (cattle, pigs, poultry, goats, sheep) or in rodents and/or small mammals in farm environments. Where prevalence ranges were reported, the mean value of the range was used. For the pathogens and parasites that were considered in multiple publications the mean value and corresponding standard error were calculated.

## 3 Results

### 3.1 Search process

**Fig. 1:**
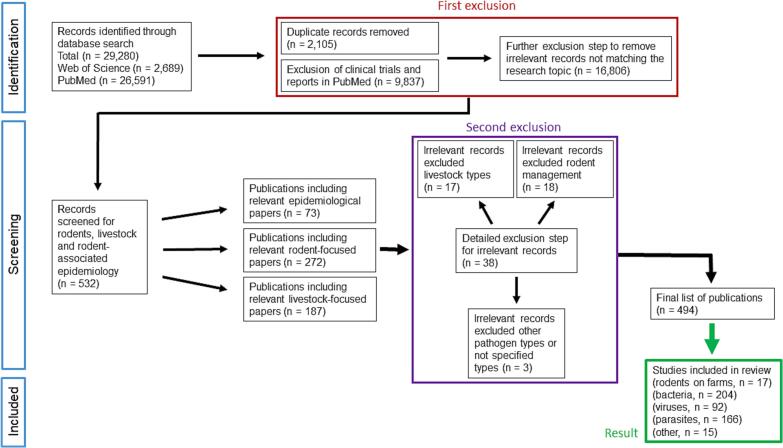
Literature search process to retrieve relevant articles for the review. Indicated are the steps ‘Identification’, ‘Screening’ and ‘Included papers’ used to narrow down number of articles according to the inclusion/exclusion criteria.

An initial search of two scientific databases resulted in 29,280 publications (Figure 1). After removing duplicates (n = 2,105) purely clinical publications (n = 9,837) and irrelevant publications (n = 16,806), 532 research articles remained. Interestingly, PubMed yielded many more hits (n = 26,591) than Web of Science (n = 2,689) with the identical search term. The results in PubMed contained many more publications with a medical, microbiological and epidemical model focus that were not covered by the inclusion criteria. The resulting 532 records were reduced in the second exclusion step by thorough research of the abstracts to exclude irrelevant studies. Certain livestock types like camel, horse, fish, goat and sheep were excluded (n = 17) and records dealing with rodent management (n = 18) and other pathogen types (n = 3) were eliminated to finally obtain 494 articles that meet all relevant criteria.

### 3.2 Pathogens

**Fig. 2:**
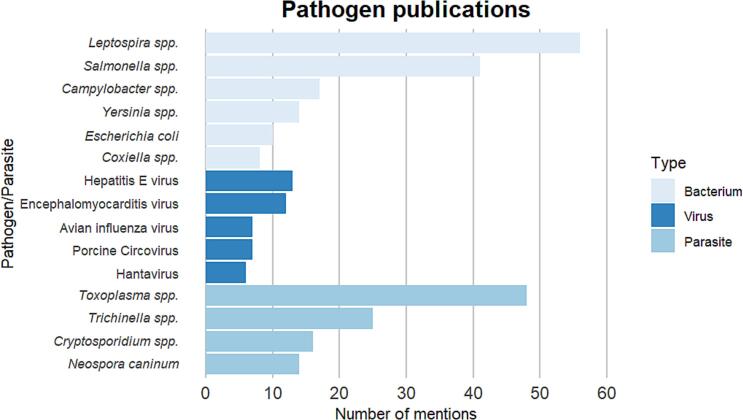
Number of mentions of the most relevant pathogens and parasites in the literature found during the search process.

A total of 77 different pathogens and parasites were addressed in the study-relevant publications. Among this number, the distribution was relatively even for bacteria (34%) viruses (30%), and parasites (31%). Other types of pathogens were rare (5%). There were many more studies that investigated zoonotic pathogens (72%) than non-zoonotic pathogens (14%) or other pathogens where the zoonotic potential is unclear (14%). In this final number of relevant articles, 204 articles considered bacterial pathogens as main topic, 92 focussed on viruses and 166 on parasites. Other articles dealt with pathogens not classified into these three groups (n = 15) as well as studies of rodents on farms that did not specify the pathogens (n = 17). Among the bacteria, most publications investigated *Leptospira* spp. (n=56), *Salmonella* spp. (n = 41), *Campylobacter* spp. (n = 17), *Yersinia* spp. (n = 14), *Escherichia coli* (n = 10) and *Coxiella* spp. (n = 8) (Figure 2). Viruses mentioned most often were Hepatitis E virus (n=13), Encephalomyocarditis virus (n = 12), Avian influenza virus (n = 7), Porcine Circovirus (n = 7) and Hantavirus (n = 6). Publications about parasites mostly dealt with *Toxoplasma* spp. (n = 48), *Trichinella* spp. (n = 25), *Cryptosporidium* spp. (n = 16) and *Neospora caninum* (n = 14).

Based on the literature search of this high number of research articles in the study-relevant topic of rodent-borne diseases, a detailed list of all pathogens found has been compiled divided into bacteria (supplemental table 1), viruses (supplemental table 2), parasites (supplemental table 3) and further types (supplemental table 4). Within the table, the classification, disease name, zoonotic potential, and main target hosts are specified. The list can be found in the appendix to this review article.

### 3.3 Rodents

**Fig. 3:**
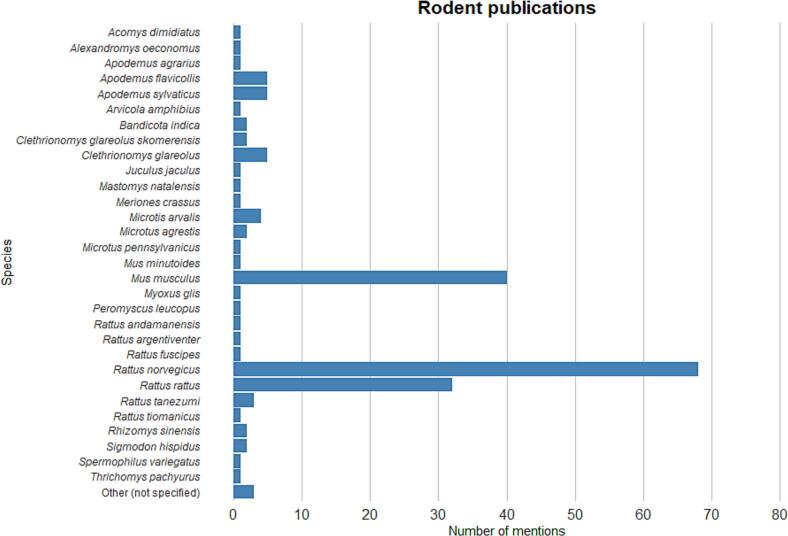
Number of mentions of the most relevant rodent species in the literature found during the search process.

Commensal rodents such as brown rats (n = 68), roof rats (n = 32) and house mice (n = 40) are most frequently mentioned (Figure 3). In addition, there are publications on *Apodemus spp.* (n = 12), *Clethrionomys glareolus* (n = 6) and *Microtus spp.* (n = 6). Other species studied are mentioned only sporadically.

### 3.4 Pooling

Data pooling revealed the highest mean prevalence (with at least two reports) in cattle (n_total_ = 45) for *Leptospira* spp. (mean = 23.78 ± 4.49, n = 19), *Toxoplasma* spp. (mean = 23.58 ± 8.69, n = 4), and *Neospora caninum* (mean = 21.13 ± 7.74, n = 6) (supplemental table 5). In pigs (n_total_ = 61) prevalence was highest for Encephalomyocarditis virus (mean = 54.17 ± 12.16, n = 3), *Clostridium* spp. (mean = 51.85 ± 7.23, n = 2), and *Leptospira* spp. (mean = 45.75 ± 9.08, n = 7). In poultry (n_total_ =18) *Campylobacter* spp. (mean = 52.90 ± 9.87, n = 5), Dermatophytes (mean = 43.15 ± 38.45, n = 2), *Salmonella* spp. (mean = 24.67 ± 7.40, n = 3), and *Toxoplasma* spp. (mean = 22.64 ± 10.94, n = 2) prevalence were highest. For sheep (n_total_ = 8) and goats (n_total_ = 12), it was *Toxoplasma* spp. (sheep: mean = 63.21 ± 1.25, n = 2; goats: mean = 54.34 ± 17.58, n = 3) and *Leptospira* spp. (sheep: mean = 21.66 ± 13.87, n = 4; goats: mean = 19.34 ± 9.29, n = 6). Among rodents and other small mammals (n_total_ = 103), *Lawsonia* spp. (mean = 63.96 ± 10.44, n = 3), *Campylobacter* spp. (mean = 37.00 ± 4.00, n = 2), *Bartonella* spp. (mean = 34.44 ± 12.93, n = 4), *Staphylococcus* spp. (mean = 31.47 ± 21.63, n = 3), ectoparasites (mean = 31.25 ± 13.38, n = 4), *Clostridium* spp. (mean = 31.00 ± 4.95, n = 3), and helminths (mean = 29.15 ± 8.40, n = 9) showed the highest mean prevalences.

## 4 Discussion

Rodents can be reservoir hosts of various pathogens that can be dangerous for livestock, pets and humans. Their mobility, reproduction cycle and often close association to farm environments define the perfect role of rodents in the transmission process of pathogens. Rodents often find direct access to livestock, as their feed and housing offers attractive living conditions. This leads to increased direct contact between rodents and farm animals, which also increases the risk of contaminated food being ingested.

### 4.1 Pathogens

#### 4.1.1 Rodents and bacteria

##### 4.1.1.1 Leptospira

The most scientific publications on rodent-borne bacterial pathogens in livestock farming mention *Leptospira* spp. The zoonotic diseases caused by multiple species of this gram-negative spirochete are of zoonotic relevance [31]. Leptospirosis occurs worldwide with a high epidemic potential in countries with humid subtropical and tropical climates [31], [47]. The World Health Organization through the Pan American Health Organization estimates more than 500,000 cases of leptospirosis each year [48]. Disease outbreaks cause high economic losses especially in pig farming as reproductive failure and underdeveloped piglets [32]. Human infection leads from flu-like symptoms up to kidney issues and pulmonary haemorrhages with significant mortality [31]. A large number of wildlife species can be infected with *Leptospira* including rodents and other small mammals that are the most significant carriers of the pathogen [33]. Different rodent species act as reservoir hosts for different *Leptospira* serovars. The reservoir hosts spread the pathogen into the environment through their excretions, blood or saliva [32]. A direct transmission through bites is possible but an indirect transmission through contaminated water, food or fluids is more frequent [31]. In Europe, wood mouse (*Apodemus sylvaticus*), yellow-necked mouse (*Apodemus flavicollis*) and bank vole (*Clethrionomys glareolus*) are carriers of various types of *Leptospira* [33], [49], [50], [51]. In other species such as striped field mouse (*Apodemus agrarius)*, common vole (*Microtus arvalis*) and field vole (*Microtus agrestis*), only one *Leptospira* species has been detected so far [33]. Globally, there is ample evidence of the prevalence of *Leptospira* in rodents, especially in genera of *Rattus, Mus, Akodon*, and *Oligoryzomys* [48], [52], [53], [54], [55], [56].

##### 4.1.1.2 Salmonella

Salmonellosis represents the second most reported foodborne bacterial gastrointestinal infection in humans after campylobacteriosis causing over 60,000 cases in the European Union (EU) in 2021 [40]. Within the EU, the European Food Safety Authority (EFSA) estimates an overall economic burden of €3 billion a year [57]. The gram-negative bacterium *Salmonella* (family: Enterobacteriaceae) has more than 2,300 serotypes and is extremely persistent [58]. Birds and mammals are generally known as main reservoir for *Salmonella*, and can transmit bacteria in their intestinal tracts, mostly without showing any clinical symptoms [59]. The zoonotic disease can be transmitted directly or indirectly between animals and humans through contaminated food or water and by contact with infected individuals [57]. Meat products (chicken, cattle) are the source for most cases of *Salmonella* infections, in particular chicken meat and egg products are known to be reservoirs due to the ability of *Salmonella* spp. to proliferate in the gastrointestinal tract of poultry [41]. Rodents carry *Salmonella* most importantly commensal species such as roof rats and house mice [41], [42], [60]. As reservoir hosts, they introduce, amplificate and spread specific *Salmonella* serotypes in commercial layer hen farms [43], [58], [60], [61]. Shifting of predominant serotypes in roof rats was demonstrated in a monitoring study [41] and suggests that the rodents’ role should be upgraded from being a reservoir host into a shifting driver of multiple serotypes. The prevention of salmonellosis therefore requires not only a correct handling of food but also a comprehensive approach and methodology at farm level to keep rodent presence on a low level.

##### 4.1.1.3 Campylobacter

Campylobacteriosis is the most frequently reported zoonosis in the EU [59] and is responsible for high economic losses in productivity with an estimated value of around €2.4 billion a year [62]. *Campylobacter spp.* is a small, spirally curved, gram-negative bacterium with *Campylobacter jejuni* (95% of infection cases) and *Campylobacter coli* (5%) [63], [64] as the most relevant pathogens. Campylobacteriosis is a zoonotic disease transmitted directly or indirectly between animals and humans, mainly on a foodborne route [62]. Pathogenic bacteria from the *Campylobacter* genus are thermophilic and able to survive for several weeks in food products stored at low temperatures [65], [66]. Multiple food-producing livestock species are carriers of *Campylobacter*, especially undercooked poultry but also pig and cattle meat is considered as main source for human infection [63]. Wild rodents can spread the bacteria between farms [63], [64], [67], [68] with higher risk in organic farms where contact with livestock is more likely and rodenticides are used less frequently [69]. Previous studies have identified a possible link between the presence of rodents or the lack of rodent control and a higher prevalence of *Campylobacter* in chicken flocks [69], [70], [71], [72]. In several rodent species such as *Rattus* spp. and *Mus* spp., *Campylobacter* has been identified in their intestinal tracts [73]. In particular, house mouse and brown rat are species that commonly live in close proximity to humans [63] leading to increased transmission risk, especially during the colder season.

##### 4.1.1.4 Escherichia coli

*Escherichia coli* is part of the normal bacterial flora present in the intestines of healthy humans and animals. However, some *E. coli* strains such as STEC (shigatoxigenic *Escherichia coli*)/VTEC (verocytotoxin-producing *Escherichia coli*) belong to a pathogenic group that can cause serious infections (diarrhoea) in humans [74], wild and domestic animals [75], [76], [77], [78]. Humans get infected with VTEC by consuming or handling contaminated food (raw milk products, undercooked meat) or water or through direct contact with infected animals [74]. Avian colibacillosis caused by avian pathogenic *E. coli* (APEC) has a considerable impact on the poultry industry worldwide [79], [80] as high morbidity and mortality lead to high economic losses [81]. Infection causes multiple organ lesions and both the broiler and layer farms are affected [82]. Commensal rodents such as brown rats have the potential to carry and transmit *E. coli* [83], [84]. Interaction among peridomestic rodents, livestock, humans and the environment on farms can facilitate spreading pathogenic *E. coli* [85].

##### 4.1.1.5 Yersinia

*Yersinia* spp. includes the three well known human and animal pathogenic *Yersinia pestis* (etiological agent of plague), *Yersinia enterocolitica*, and *Yersinia pseudotuberculosis*. Yersiniosis represented the third most commonly reported foodborne gastrointestinal infection in humans in the EU according to the 2022 Zoonoses report of EFSA. In 2022, *Yersinia enterocolitica* causes the majority (98.7%) of human cases, *Yersinia pseudotuberculosis* only 1.3% [86]. Both pathogens are widespread in the environment and the epidemiological mechanisms are complex and not yet fully understood [87]. The source of yersiniosis is contaminated food, especially pork products [88] but *Yersinia* spp. can be isolated from different animals and food, as well as from water, plant, soil that is contaminated with faeces of infected animals [89]. A recent study revealed that rodents may be carriers of more than one strain of *Yersinia enterocolitica* [87]. Rodents such as house mice are a considerable factor in *Yersinia pseudotuberculosis* transmission on pig farms [90]. Especially house mice but also voles are caught frequently in farm environments [91] and *Yersinia enterocolitica* have been isolated from intestinal samples of commensal rodents [87].

##### 4.1.1.6 Q fever

Q fever, also known as coxiellosis in animals, is a zoonotic disease that is present worldwide (exception: New Zealand, Norway and Iceland) [92], [93] caused by the strictly intracellular, gram-negative bacterium *Coxiella burnetii* [94], [95], [96], [97] affecting both humans and animals. The spore-like forms are highly resistant to harsh environmental conditions [92], the bacterium can survive for years in the environment and is able to travel long distances via aerosols [98]. Inhalation of aerosolised bacteria and direct contact with contaminated products are primary infection routes for humans besides gastro-intestinal and human-to-human transmission [99], [100]. In livestock, clinical signs for coxiellosis in domestic ruminants such as goats, sheep and cattle are abortion, stillbirth and small or weak offspring [92]. Commensal brown rats seem to be an important factor circulating and spreading *Coxiella burnetii* to domestic animals and humans [101]. Prevalences in wild rodents and shrews [102] and in commensal rat species are high [95], [96]. Additionally, *Coxiella* was identified in a gerbil species (*Meriones shawi*) and its ticks [102] and in house mice (*Mus domesticus*) [103].

#### 4.1.2 Rodents and viruses

##### 4.1.2.1 Hepatitis E virus

Most scientific publications considered Hepatitis E virus (HEV), a single-stranded RNA virus (family: *Hepeviridae*) that is a leading cause of viral hepatitis globally with millions of cases annually [12], [104], [105]. Most reports of human infections were due to Orthohepevirus A genotype 3 (HEV-3) as zoonotic origin with a wide host range linked to the pig meat industry and products [12]. Rodents are susceptible to infection with a diverse range of HEV species and represent a potential infection risk for humans and livestock [106], [107]. Brown rats carry a distinct HEV species, Orthohepevirus C (ratHEV), also known as *Rocahepevirus ratti*, that was first detected in 2010 [108] and can cause severe chronic hepatitis in humans [109], [110], [111]. RatHEV was previously not thought to be zoonotic until the first case of human infection in Hong Kong [112] and since then further infections surfaced [113], [114]. Roof rat and brown rat are the main reservoirs of ratHEV and direct or indirect contact have been suggested as potential transmission route [112], [115], [116]. However, other species such as greater bandicoot rat (*Bandicota indica*) and several *Rattus* species (*R. tanezumi*, *R. flavipectus*, *R. losea*) carry ratHEV [12], [106], [115], [116], [117], [118], [119]. Since the first human cases, the number of infections has increased in Europe over the last years [115].

##### 4.1.2.2 Encephalomyocarditis virus

The Encephalomyocarditis virus (EMCV) is a small, single-stranded RNA virus (genus: Cardiovirus; family: *Picornaviridae*) with two known serotypes [120] that is present worldwide and infects a variety of vertebrate hosts with zoonotic potential for humans [120], [121], [122], [123]. EMCV is generally considered to cause fatal myocarditis in pigs and the course of the infection in pigs is affected by the strain virulence, exposure intensity, individual susceptibility and immune status [124]. Pigs are likely impacted severely [125], [126], however, EMCV can cross the species barrier (multi-host dynamics) [122] and the possible transmission to humans has increased the sensitivity for the virus [121]. Two main mechanisms of transmission are currently suggested in domestic pigs (I) oral infection of faeces/urine of infected rodents through contaminated food and water or (II) pig-to-pig transmission via excretion or transplacental transmission [125], [127], [128]. Rodents, especially commensal rodents such as brown rats and house mice, are considered natural hosts, where EMCV usually persists without causing symptoms [127].

##### 4.1.2.3 Avian influenza virus

Influenza A viruses (IAV) pose one of the major health risks to the poultry industry causing outbreaks in poultry and wild birds and severe human infections in multiple countries [129], [130], [131], [132]. IAVs have been isolated from a variety of marine and terrestrial mammals (including humans) and a wide range of birds and represent one of the most important zoonotic pathogens that continue to evolve and challenge animal and human health [133], [134], [135], [136], [137]. A majority of the studies have focussed on the highly pathogenic avian influenza virus (HPIAV) compared to the low pathogenic subtype (LPIAV) because of the threat of HPIAV to animal and public health as well as economics [138]. In most cases, IAV is introduced into domestic poultry through direct or indirect contact with infected wild birds [139]. Open outdoor areas in free-range poultry systems are therefore a high-risk factor for IAV transmission from wild birds to commercial poultry [140], [141]. Further potential reservoirs of IAV could be commensal rodents in the surroundings of farms [142], [143], [144]. The role of rodents in the transmission route of IAV outbreaks is not fully identified. It seems likely that fur or paws of rodents can transport virus material but there are no published data on the survival time of the virus on rodents [140]. However, recent study results suggest that wild rats might play some role as reservoirs or mechanical vectors of IAV circulation [145].

##### 4.1.2.4 Porcine circovirus

Porcine circoviruses (PCVs) belong to the Circovirus genus (family: *Circoviridae*) including the three species PCV1 (apathogenic), PCV2 (pathogenic), and PCV3 (pathogenic) [146]. PCV2 and PCV3 have gained strong interest in the swine industry as they were confirmed as causal pathogen for the porcine disease post-weaning multisystemic wasting syndrome (PMWS) [147], [148], porcine dermatitis and nephropathy syndrome (PDNS), reproductive failure and other syndromes [149], [150]. In pig farms, PCV3 can infect animals of different ages and productive stages [151], [152]. Domestic pigs and wild boar are generally considered as natural reservoirs of PCV2 [153] and PCV3 [151]. Only a few studies have confirmed PCV infections in field rodents [146] but reports show that PCV2 can frequently spillover from pigs to rodents on farms [154], [155], [156] and that it has the capacity to naturally infect rats [157]. Recent findings could not confirm that rodents have an actual role as PCV reservoirs [157], [158] but further investigations should elucidate their potential as carrier.

##### 4.1.2.5 Hantavirus

Hantaviruses are single-stranded RNA viruses (genus: Orthohantavirus; family: *Hantaviridae*) containing more than 50 known species [159], [160] and establish persistent infections in their reservoir mammalian hosts such as rodents, bats and insectivores [161], [162]. Rodents are the natural hosts of most hantaviruses and each virus species is usually associated with one specific rodent species [163], [164], mainly of the families *Cricetidae* and *Muridae* [165]. These reservoir hosts maintain the infection without developing clinical signs [166] and disseminate the virus via urine, faeces and saliva [22]. Through inhalation of aerosolised viruses from these excretions, humans become infected [167], especially farm workers are at risk due to the high potential for encounters with wild and commensal rodents. The most common and widespread hantavirus in Europe is Puumala virus (PUUV) [163], which is associated with a mild form of haemorrhagic fever with renal syndrome (HFRS) [167] whereas Hantavirus pulmonary syndrome (HPS caused by representatives of *Orthohantavirus* genus) is mostly reported in the Americas with high human mortality [168]. In certain areas in Argentina, increased prevalences for hantaviruses causing hantavirus pulmonary syndrome were detected among rodents resulting in higher incidence in humans than in other regions [169]. *Oligoryzomys longicaudatus* has been clearly established as a reservoir for Andes hantavirus in South America. Transmission within rodent populations via direct contact has been demonstrated experimentally [170] as has transmission to humans through zoonotic spillover events [171]. Other important reservoir species include *Sigmodon hispidus* (Ozark virus) [172], *Oligoryzomys flavescens* (Lechiguanas virus) [173], *Peromyscus maniculatus* (Sin Nombre virus) [174], and genera such as *Akodon* [175] and *Necromys* [176]. The Seoul virus was first reported in Great Britain [177] and there have been several reports of human disease and hantavirus infection in brown rats [160]. They are considered as SEOV carrier for the global dissemination [178]. The rarely human pathogenic Tula virus (TULV) is mainly associated with the common vole (*Microtus arvalis*) but was also detected in field voles (*Microtus agrestis*) and water voles (*Arvicola* spp.) [163]. In addition, in European rodents there are Dobrava-Belgrade hantavirus (DOBV), Saaremaa hantavirus (subspecies of DOBV) and Tatenale hantavirus (TATV) [165], [179].

#### 4.1.3 Rodents and parasites

##### 4.1.3.1 Toxoplasma

Toxoplasmosis, caused by the ubiquitous protozoan parasite *Toxoplasma gondii* [180], [181], [182] is a globally distributed zoonotic food-borne disease that affects humans and other warm-blooded vertebrates such as cats [183]. The parasite has a complex life cycle whereby Felids have been identified as definitive hosts sharing oocysts to the environment via their faeces after primary infection [181]. Birds, rodents and also livestock can act as intermediate hosts and humans can get infected through poor hygiene, consumption of raw meat, contaminated food and water, transplacental or by blood transfusions and organ transplantation [184]. Toxoplasmosis is responsible not only for global health risk but also for high economic losses due to genital and fertility disorders (mummification, stillbirth, abortion) in farm animals [185]. The risk for pigs is associated with the presence of cats and rodents, and their accessibility to pig food and water stations [184], [185], [186], [187], [188]. Free-ranging poultry also play a role as intermediate host of *Toxoplasma gondii* and is a primary source of infection for humans and cats [189]. In goats, infections can cause significant reproductive disorders and systemic disease in rare cases [190]. Rodents such as brown rat and roof rat [180] and field mouse [191] are carriers of *Toxoplasma gondii* and are potential intermediate hosts [192] getting infected due to ingestion of sporulated oocysts [193]. *T. gondii* infections in rodents can be of epidemiological importance, as rodents can serve as a source of tissue cysts for *Felidae* [194], [195].

##### 4.1.3.2 Trichinella

Trichinosis is a parasitic disease caused by nematode *Trichinella spiralis* as etiological agent and maintains in a domestic cycle that includes pigs, rodents and other wild animals such as wild boar (*Sus scrofa*) and red foxes (*Vulpes vulpes*) [196], [197]. Trichinosis is classified as zoonotic disease [198] with a high risk for humans. Infection is usually via the ingestion of muscle tissue containing live *Trichinella* larvae [199]. However, humans are accidental hosts that acquire infection through the consumption of raw or undercooked meat infected with encysted *Trichinella* larvae [200]. In the swine industry, infection is favoured by poor sanitary practices, inadequate nutrition, and substandard pig management [201]. Pigs are reservoirs and potentially introduce the parasite into domestic environments [198]. Transmission can also occur via reservoir hosts such as brown rat, especially on pig farms without sufficient rodent control measures and a low level of sanitary conditions [202]. Studies revealed high prevalence of *Trichinella* in rat species on farms [199], [203], [204], [205] suggesting circulation of the pathogen in wild and commensal rodents.

##### 4.1.3.3 Cryptosporidium

The protozoan parasite *Cryptosporidium* is the pathogenic agent responsible for the gastrointestinal disease cryptosporidiosis in humans and is considered as a public health concern, especially in developing regions around the world [206], [207], [208], [209], [210]. In addition, *Cryptosporidium* is prevalent in a variety of domestic and wild animals [211]. Transmission takes place through the faecal-oral route, directly between humans or through contact with infected animals, or indirectly via ingestion of contaminated food/water [212], [213]. Most studies of animal cryptosporidiosis have focused on farm animals (pigs, sheep and cattle) or pets [214]. There are >40 species and genotypes of *Cryptosporidium* in rodents, with 17 genotypes found in *Rattus* genus [215], [216], [217], [218]. Both the murid and cricetid families include synanthropic species that could be important reservoirs of *Cryptosporidium* infections [218]. The parasite was first detected in brown rat’s faeces in Japan [219].

##### 4.1.3.4 Neospora caninum

Neosporosis is caused by the protozoan *Neospora caninum* (Apicomplexa, family: *Toxoplasmatidae*) and is a serious parasitic disease in dairy cattle and dogs worldwide [220], [221]. The economic loss due to *Neospora*-associated abortions in cattle are extremely high with annual cost of more than one billion US$ [222], [223]. Domestic and wild dogs are definitive hosts, whereas multiple species are intermediate hosts of the parasite, including pigs [221]. Antibodies in humans have been detected, however, the zoonotic potential is uncertain [224]. Contamination of the environment by infected dogs through oocysts in their faeces and the presence of tachyzoites in the placenta of aborted cattle may result in human exposure [225]. It should be noted that dairy cattle are more susceptible to neosporosis compared to beef cattle [226]. Rodents are known for their status as intermediate host for *Neospora caninum* and could play a significant role in maintaining the life cycle of the parasite [222]. Recent studies revealed that the presence of rodents in dairy farms shows a positive correlation with the *Neospora caninum* prevalence in cows [227]. This is especially the case for commensal species such as roof rats [228]. However, further research is needed to determine the exact role and influence of rodent populations on outbreaks of neosporosis.

#### 4.1.4 Rodents and other pathogen types

In addition to the well-known groups of pathogens - bacteria, viruses, and parasites - there are other groups, such as fungi and prions, that have zoonotic potential and can cause disease in livestock. These pathogen types are underrepresented in the results of the literature search, but should not go unmentioned.

##### 4.1.4.1 Dermatophytosis

Dermatophytosis, caused by zoonotic fungi group dermatophytes is considered as public health issue in many countries [229]. The most common isolated pathogens in animals with dermatophytosis are *Trichophyton mentagrophytes*, *Microsporum gypseum*, and *Microsporum canis* [230]. Rabbits and rodents can be infected with different types and act as carriers via different structures such as hair, body surface or skin [229]. It is suggested that ectoparasites can also play an important role in transmission of dermatophytosis [231], but information about the epidemiology in rodents is limited.

##### 4.1.4.2 Microsporidiosis

*Enterocytozoon bieneusi* is a zoonotic microsporidian species responsible for microsporidiosis, causing a high number of human infections, especially in China [232]. *E. bieneusi* has a large range of hosts including various animals such as cattle [232] and wild rodents [233] and is transmitted typically through ingestion of spore contaminated food and water or contact with infected animals [234], [235]. There are about 60 different genotypes present in rodents [235] showing their major role in transmission. In farm environments, *E. bieneusi* is prevalent in commensal rodents carrying zoonotic genotypes and acting as natural reservoirs [235] leading to an increase in zoonotic risk for livestock and humans.

##### 4.1.4.3 Prion disease

Prions cause transmissible spongiform encephalopathies (TSE), is a chronic disease that attacks the nervous system associated with high mortality in humans and livestock. Few studies have focused on the pathogenesis of TSE in natural hosts such as sheep and goats [236], [237]. In cattle, prions cause bovine spongiform encephalopathy (BSE), a disease that received much attention in the past [238]. Contrary to BSE in cattle, TSE is an endemic disease meaning that the disease agent does transmit among sheep under natural conditions [238] through direct contact or contamination in the environment [238]. Nothing is yet known about the potential role of rodents in transmission. To date, there is no conclusive epidemiological evidence that rodents play a significant role as natural vectors of TSE in livestock. Nevertheless, is mentioned here in light of the uncertain role of rodents in transmission dynamics.

### 4.2 Further issues

In addition to the health aspects, which pose a significant problem for livestock and farm workers (due to the frequent zoonotic potential of pathogens and parasites), the economic losses associated with the diseases are also of great importance. Recent studies show that zoonoses lead to nearly 2.5 billion cases of illness and 2.7 million deaths annually [239]. Zoonotic events cause high economic damage exceeding USD 100 billion per outbreak for some diseases such as avian influenza, which particularly affects low- and middle-income countries [239]. Diseases can lead to reduced productivity of livestock due to illness-related performance losses, increased costs for veterinary treatment, diagnostics, and biosecurity measures, as well as disruptions in supply chains [240]. Additionally, export bans following outbreaks have a negative impact on trade [239].

Certain rodent genera cause the greatest economic losses worldwide with rat and shrew species particularly often infected with hantaviruses and *Bartonella* [241]. *Apodemus* and *Microtus* species are well known for their high potential as hantavirus [163] and *Leptospira* [242] carriers in Europe, *Akodon* and *Oligoryzomys* species as *Leptospira* carriers in the Americas [55], [243]. Many pathogens show specificity for certain rodent reservoirs [15], which is important for risk prediction and prevention [241] and these taxa are particularly “expensive” in terms of economic losses. Rodents (especially genera of *Rattus*, *Myodes* and *Microtus*) act as permanent reservoir hosts for numerous pathogens that can infect livestock [244]. Their role in transmission can vary and depends on the pathogen. Rodents themselves usually do not show symptoms, but they chronically excrete pathogens in their urine, faeces, or saliva and therefore represent a continuous source of infection for farm animals [245]. Within the framework of the One Health rodents matter for antimicrobial resistance (AMR) as vectors or intermediaries within an interconnected system comprising livestock, the environment, and humans [246]. In this context, rodents primarily act as ecological vectors and disseminators of resistance by carrying resistant microorganisms and resistance genes (ARGs) in their intestines, on their skin, or within parasites [84], [247]. However, these are not necessarily transmitted as pathogens, but often as genetic information between ecological compartments. The key factor here is horizontal gene transfer (HGT) as a mechanism for the spread of resistance [84]. Therefore, rodents are not a primary source of resistance, but rather ‘mobile vectors’ that facilitate the spread of resistance between reservoirs.

Additionally, spatial movement of commensal rodents play a crucial role in pathogen transmission and recent ecological investigations in a farm environment showed that rodents can adapt their home range size and movement behaviour to the prevailing situation [248]. The rodents access stables, feed storage areas, or drinking systems, where they cause contamination [243], [249]. Although rodents rarely move between different barn buildings [248], even small movements can cause disease outbreaks among livestock. Contact with carcasses also plays a major role, especially in pig farming. This is particularly relevant in intensive livestock farming, farms with poor biosecurity, and in tropical and subtropical regions (but also in Europe).

Biosecurity is defined as a strategic, integrated approach within One Health to risk mitigation that addresses plant, animal, environmental, and human health collectively [250]. Diseases in livestock affect food quality, the availability of safe animal products, and the income of people who work in livestock industry. The WHO guidelines emphasize that animal health, animal welfare, and human health must be protected together because harm to animals has direct economic and health consequences for humans [251]. Rodent control should be considered as an important part of good biosecurity on farms in order to reduce disease risks, as rodents can introduce or spread infectious agents on agricultural properties [27]. Modern rodent control should follow the ‘Ecologically based Rodent Management’ (EBRM) principle, which involves combining multiple measures while minimizing the use of chemicals [252]. EBRM is an evidence-based approach comprising (1) prevention, (2) monitoring, (3) intervention with techniques that are effective, consider ecological and economical as well as social aspects, and (4) evaluation and adaption of management [253]. Special measures such as structural sealing (infrastructure), feed and waste management (closed silos, no open feed residues), and environmental management (vegetation control) are necessary and crucial. During the monitoring stage, infested areas are inspected for traces and feeding damage, and various monitoring methods (e.g., camera traps) or digital tools (e.g., sensors) are used. Intervention involves the use of mechanical traps and barriers, or chemical agents (minimal use). Finally, as part of the evaluation, the effects are documented and the strategies are adjusted. EBRM, also considered as an application of ‘Integrated Pest Management’ (IPM), is a key element in implementing the One Health approach in agriculture [254]. Prevention is more effective and sustainable than purely chemical approaches [255]. Biosecurity must be approached in a systemic and cross-sectoral manner to effectively reduce disease and pest risks [256]. There is still enormous potential for improvement and more effective approaches, particularly in low- and middle-income countries, where health care for humans and livestock can be poor and some tools for rodent management unavailable or unknown.

For some rodent-pathogen-human systems there is a clear association of rodent density, pathogen prevalence and human incidence (PUUV, [257], [258]) but little is known about such relationships in livestock. Appropriate rodent management can have a direct impact on the incidence of zoonotic diseases in humans such as cutaneous leishmaniasis, where an interruption in rodent management leads to an increase in human cases and recommencement leads to a decline [259]. However, for many other pathogens/diseases the drivers of human and let alone livestock infection and the effect of rodent management it is unknown. A decline in rodent population size alone does not guarantee a reduction in pathogen prevalence, as species composition, biodiversity, and ecological networks are also crucial [260]. Rodent management may even cause an opposite effect when the removal of individuals/populations/species leads to an influx of other individuals/populations/species carrying pathogens that were previously absent. Rodent management measures should work synergistically with environmental and biodiversity management in order to control pathogens holistically.

Climate change can affect the transmission of pathogens from rodents to livestock in various ways and plays a major role in the One Health approach. It can affect the reproduction, survival rate, and distribution of rodents [261], [262]. The risk of pathogen transmission may change as a result, as higher temperatures increase the replication rate of viruses and bacteria in rodents, and climate-related stress weakens the immune system [263]. Climate-induced habitat loss or food shortages can cause rodents to move closer to farms increasing the likelihood of pathogen transmission to livestock [10]. Furthermore, changes in rodent population dynamics may occur [264] and climate change can influence the spread of vectors such as ticks or fleas, which transmit pathogens from rodents to livestock [265].

The pooling of data demonstrates high prevalence for multiple specific pathogens and parasites but there is substantial variability across pathogens and studies. This is driven by a combination of ecological dynamics, host and environmental heterogeneity, methodological differences in study design and diagnostics, and multiple sources of bias such as imperfect detection and sampling effects [266], [267], [268].

Across livestock taxa the most frequently detected pathogens are *Leptospira* spp., *Toxoplasma* spp., *Campylobacter* spp. whereas a wider range of pathogens and parasites is found among rodents and other small mammals. These particular pathogens are featured in many studies because they can infect multiple host species, are well adapted to agricultural systems and are efficiently transmitted (directly, via the environment, or via feed) [269], [270], [271]. Small mammals generally have high population densities, short generation times and close contact with each other and with the environment in and around farms. This can lead to greater pathogen diversity, but not necessarily to similar prevalences for individual pathogens [17]. Similarities in prevalence are probably due to common drivers of infection, similar epidemiological patterns and comparable management effects.

Overall, there were more studies on bacterial pathogens than on viruses and parasites. This discrepancy is probably the result of several interrelated epidemiological and methodological factors. In previous global reviews of the veterinary and animal welfare literature the proportion of bacterial pathogens (37%) is higher than that of viral pathogens (33%) or parasites (19%) [272]. This is similar for bacteria and viruses in our review. Compared to viruses and parasites, bacterial pathogens are easier and less expensive in diagnosis, cultivation, and isolation. These methodological challenges may have resulted in more publications. Research funding and prioritization may also affect the percentages. Bacterial pathogens are often notifiable have immediate implications for public health and trade. They tend to receive more research funding than viral or parasitic pathogens, which exhibit more subtle or difficult-to-measure disease patterns [5], [272], [273]. Studies of viruses and parasites are often more complex and typically require multidisciplinary approaches. More studies of parasites have emerged only over the past few years (>30%) which could be an indication that the enormous economic costs associated with parasitic disease outbreaks in livestock are driving an increase in research efforts.

When rodents, pathogens, and livestock are viewed as an integrated system, several common patterns in transmission routes, reservoir dynamics, and predisposing conditions become apparent. Rodents link wildlife to livestock farming regardless of the type of pathogen [274]. Pathogens with longer environmental stability (especially bacteria and spore-forming organisms) are more easily transmitted through indirect contact [5] than pathogens that rely on direct contact (certain viruses and parasites). In the latter there is strong density dependence and seasonal spillover peaks [275]. Management and environmental conditions strongly modulate the risk for all pathogen groups, informing the One Health approach [276].

Several critical knowledge gaps became apparent that limit the effectiveness of preventive measures, economic assessments, and risk evaluation. For instance, there often is no detailed knowledge of the precise mechanisms of pathogen spill over from rodents to livestock, particularly in for parasites and viruses that are difficult to detect [276], [277], [278]. This hampers risk assessments and intervention. Furthermore, multi-species reservoir systems and their interactions with one another and with livestock remain poorly understood [8]. The lack of data on reservoir diversity and host-animal networks makes it difficult to model transmission risks. There are further knowledge gaps in pathogen surveillance and diagnostics (particularly regarding viruses and parasites) [274], which means that early warning systems are incomplete and underreporting of spillover events is likely. Moreover, quantitative data on the economic costs of rodent-borne pathogens in livestock production are limited, making it difficult to conduct cost-benefit analyses.

Future research should focus on integrated One Health approaches, with studies combining studies rodents, livestock health, environmental conditions, and human interactions. This could support our understanding of spillover mechanisms, identify risk factors, and optimize management strategies [279]. In addition, future research should emphasize reservoir communities (rather than just individual species) and underrepresented pathogens (viruses and parasites) [280], [281]. Furthermore, it is crucial to quantify the direct and indirect economic impacts of spillover events for more accurate assessments of economic costs. This would allow economic models to be integrated into epidemiological studies.

## 5 Conclusions

In conclusion, this review highlights the substantial and multifaceted impact of rodent-borne pathogens on livestock systems within a One Health framework. Beyond their well-recognised public health implications, these pathogens impose significant economic burdens through reduced productivity, increased management costs, and disruptions to trade. Rodents function as persistent reservoir hosts and mobile ecological vectors, facilitating both pathogen transmission and the spread of antimicrobial resistance across interconnected environmental, animal, and human compartments. Their adaptability to farm environments and capacity for chronic pathogen shedding underscore their importance in disease dynamics.

Effective mitigation requires integrated, ecologically informed strategies such as ‘Ecologically Based Rodent Management’, embedded within comprehensive biosecurity systems. However, the outcomes of rodent control are complex and context-dependent, influenced by ecological interactions, biodiversity, and environmental change, including climate-driven shifts in rodent populations and vector dynamics.

Significant knowledge gaps remain, particularly regarding spillover mechanisms, multi-species reservoir networks, and the epidemiology of underrepresented pathogens such as viruses and parasites. In addition, the limited availability of robust economic data constrains accurate assessments of disease burden and intervention cost-effectiveness. Addressing these gaps through interdisciplinary, One Health-oriented research will be essential for improving risk prediction, enhancing surveillance, and developing sustainable, evidence-based management strategies that safeguard livestock health, economic stability, and public health.

## Declaration of generative AI use

The authors declare that no generative AI tools were used in the manuscript process.

## CRediT authorship contribution statement

**Florian Huels:** Writing – original draft, Visualization, Methodology, Investigation, Conceptualization. **Jens Jacob:** Writing – review & editing, Conceptualization.

## Funding information

This work was financially supported by the German Federal Ministry of Agriculture, Food and Regional Identity (BMLEH) through the Federal Office for Agriculture and Food (BLE), grant number 2821ERA22D.

## Declaration of competing interest

None.

## Data Availability

The full review literature list of the literature search as well as the resulting data used to create the graphical figures (created with RStudio in R Project version 4.5.2) are available from the authors.
