# Supplementary material for: Rodent-borne pathogens as economic and zoonotic health threat to livestock farming: a review
Source: One Health. 2026 May 16;22:101448. doi: 10.1016/j.onehlt.2026.101448 (PMC13208097; doi:10.1016/j.onehlt.2026.101448)
Supplement: Supplementary file 1 — Supplementary material [file mmc1.docx]

# Supplementary material

**Table 1:** Full list of relevant bacterial pathogens found during the search process.

| **Bacterium** | | **Phylum** | **Genus** | **Family** | **Disease** | **Zoonosis** | **Main host** |
| --- | --- | --- | --- | --- | --- | --- | --- |
| *Anaplasma phagocytophilum* | Proteobacteria | | *Anaplasma* | *Ehrlichiaceae* | Anaplasmosis | Yes | Diverse |
| *Bartonella* | Proteobacteria | | *Bartonella* | *Bartonellaceae* | Bartonellosis | Yes | Diverse |
| *Bordetella bronchiseptica* | Proteobacteria | | *Bordetella* | *Alcaligenaceae* | Bronchitis | Yes | Diverse |
| *Borrelia burgdorferi* | Spirochaetota | | *Borrelia* | *Borreliaceae* | Lyme disease | Yes | Diverse |
| *Brachyspira pilosicoli* | Spirochaetota | | *Brachyspira* | *Brachyspiraceae* | Porcine intestinal spirochaetosis | ? | Pigs |
| *Brucella* | Proteobacteria | | *Brucella* | *Brucellaceae* | Brucellosis | Yes | Cattle, sheep, goats, pigs |
| *Campylobacter hepaticus* | Proteobacteria | | *Campylobacter* | *Campylobacteraceae* | Spotty Liver Disease (SLD) | No | Chicken |
| *Campylobacter jejuni* | Proteobacteria | | *Campylobacter* | *Campylobacteraceae* | Campylobacter enteritis | Yes | Diverse |
| *Clostridium difficile* | Bacillota | | *Clostridioides* | *Peptostreptococcaceae* | Diarrheal infections | Yes | Diverse |
| *Clostridium perfringens* | Bacillota | | *Clostridiodes* | *Clostriciaceae* | Clostridial myonecrosis, Gastroenteritis | Yes | Diverse |
| *Coxiella burnetii* | Proteobacteria | | *Coxiella* | *Coxiellaceae* | Q fever | Yes | Sheep, goats |
| *Escherichia coli* | Proteobacteria | | *Escherichia* | *Enterobacteriaceae* | Diverse | Yes | Diverse |
| *Francisella tularensis* | Proteobacteria | | *Francisella* | *Francisellaceae* | Tularemia | Yes | Rodents, lagomorphs |
| *Lawsonia intracellularis* | Pseudomonadota | | *Lawsonia* | *Desulfovibrionaceae* | Proliferative enteropathy disease | No | Pigs |
| *Leptospira interrogans* | Spirochaetota | | *Leptospira* | *Leptospiraceae* | Leptospirosis | Yes | Rodents, dogs, pigs |
| *Methicillin-resistant Staphylococcus aureus* | Bacillota | | *Staphylococcus* | *Staphylococcaceae* | MRSA infection | Yes | Diverse |
| *Mycobacteria* | Actinobacteria | | *Mycobacteria* | *Mycobacteriaceae* | Mycobacteriosis | Yes | Diverse |
| *Mycoplasma* | Mycoplasmatota | | *Mycoplasma* | *Mycoplasmataceae* | Diverse | ? | Diverse |
| *Orientia tsitsugamushi* | Pseudomonadota | | *Orienta* | *Rickettsiaceae* | Scrub typhus | Yes | Diverse |
| *Salmonella* | Proteobacteria | | *Salmonella* | *Enterobacteriaceae* | Salmonellosis | Yes | Diverse |
| *Staphylococcus aureus* | Bacillota | | *Staphylococcus* | *Staphylococcaceae* | Diverse infections | Yes | Diverse |
| *Yersinia enterocolitica* | Proteobacteria | | Yersinia | Enterobacteriaceae | Yersiniosis | Yes | Pigs |
| *Yersinia pestis* | Proteobacteria | | Yersinia | Yersiniaceae | Plague | Yes | Rodents |
| *Yersinia pseudotuberculosis* | Proteobacteria | | Yersinia | Enterobacteriaceae | Far East scarlet-like fever | Yes | Wild animals |

**Table 2:** Full list of relevant viral pathogens found during the search process.

| **Virus** | **Type** | **Genus** | **Family** | **Disease** | **Zoonosis** | **Main host** |
| --- | --- | --- | --- | --- | --- | --- |
| African swine fever virus | DNA virus | *Asfivirus* | *Asfarviridae* | African swine fever (ASF) | No | Pigs |
| Akhmeta virus (AKMV) | DNA virus | *Orthopoxvirus* | *Poxviridae* | Pox diseases | Yes | Mammals, arthropods |
| Porcine reproductive and respiratory syndrome virus | RNA virus | *Betaarterivirus* | *Arteriviridae* | Porcine Reproductive and Respiratory Syndrome (PRRS) | No | Pigs |
| Borna disease virus (BDV) | RNA virus | *Orthobornavirus* | *Bornaviridae* | Borna disease | Yes | Shrew |
| Bunyavirales | RNA virus | *-* | *Arenaviridae* | Hemorrhagic fever | Yes | Diverse |
| Coronavirus | RNA virus | *-* | *Coronaviridae* | SARS, MERS, SARS-CoV-2 | Yes | Land vertebrates |
| Encephalomyocarditis virus (EMCV) | RNA virus | *Cardiovirus* | *Picornaviridae* | Encephalomyocarditis and reproductive disease | No | Pigs |
| Turkey corona virus | RNA virus | *-* | *-* | Poult enteritis mortality syndrome (PEMS) | ? | Turkeys |
| Hepatitis E virus (Orthohepevirus A) | RNA virus | *Orthohepevirus* | *Hepeviridae* | Hepatitis E | Yes | Vertebrates |
| Influenza A virus (H5N1), (H5N6), (H9N2), (H3N8) | RNA virus | *Alphainfluenzavirus* | *Orthomyxoviridae* | Avian influenza | Yes | Birds |
| Influenza A virus (IAV) | RNA virus | *Alphainfluenzavirus* | *Orthomyxoviridae* | Flu | Yes | Diverse |
| Mastadenovirus C (bovine, humane, porcine) | DNA virus | *Mastadenovirus* | *Adenoviridae* | respiratory, gastrointestinal, eye infections | ? | Diverse |
| Murid gammaherpesvirus 68 (MuHV-68) | DNA virus | *Rhadinovirus* | *Orthoherpesviridae* | B-cell lymphoma, Kaposi's sarcoma | Yes | Mice |
| Orthoflavivirus | RNA virus | *Flavivirus* | *Flaviviridae* | West Nile fever, Dengue fever, Encephalitis, Yellow fever, Zirka fever | Yes | Vertebrates |
| Orthohantavirus | RNA virus | *Orthohantavirus* | *Hantaviridae* | Hantavirus pulmonary syndrome (HPS) | Yes | Rodents |
| Picornavirus Senecavirus A | RNA virus | *Senecavirus* | *Picornaviridae* | - | No | Pigs |
| Porcine Circovirus-1 (PCV1) | DNA virus | *Circovirus* | *Circoviridae* | - | - | - |
| Porcine Circovirus-2 (PCV2) | DNA virus | *Circovirus* | *Circoviridae* | Postweaning multisystemic wasting syndrome (PMWS), Porcine Dermatitis and Nephropathy Syndrome (PDNS) | No | Pigs |
| Porcine Circovirus-3 (PCV3) | DNA virus | *Circovirus* | *Circoviridae* | ? | ? | Pigs |
| Rabies virus (RABV) | RNA virus | *Lyssa* | *Rhabdoviridae* | Rabies | Yes | Diverse |
| Rift Valley fever phlebovirus | RNA virus | *Phlebovirus* | *Phenuiviridae* | Rift Valley fever | Yes | Ruminants |
| Seoul virus (SEOV) | RNA virus | *Orthohantavirus* | *Hantaviridae* | fever, kidney injury, hepatitis, gastroenteritis | Yes | Rats (*Rattus sp.*) |
| Vaccinia virus | DNA virus | *Orthopoxvirus* | *Poxviridae* | Vaccinia infections | Yes | Diverse |

**Table 3:** Full list of relevant parasitic pathogens found during the search process.

| **Parasite** | **Type** | **Genus** | **Family** | **Disease** | **Zoonosis** | **Main host** |
| --- | --- | --- | --- | --- | --- | --- |
| *Blastocystis* | Eukaryotic | *Blastocystis* | *-* | Diarrhea, gastrointestinal problems | Yes (?) | Diverse |
| *Cryptosporidium* | Eukaryotic | *Cryptosporidium* | *-* | Cryptosporidiosis | Yes | Mammals |
| *Cysticercus* | Cestode | *Taenia* | *Taeniidae* | Cysticercosis | Yes | Diverse |
| *Echinococcus multilocuraris* | Cestode | *Echinococcus* | *Taeniidae* | Echinococcosis | Yes | Mammals |
| *Eimeria spp.* | Eukaryotic | *Eimeria* | *Eimeriidae* | Coccidiosis | No | Vertebrates |
| *Enterocytozoon bieneusi* | Eukaryotic | *Enterocytozoon* | *Enterocytozoonidae* | Cholangitis, Diarrhoe | Yes | Diverse |
| *Fasciola hepatica* | Helminth | *Fasciola* | *Fasciolidae* | Fasciolosis | Yes | Herbivores |
| *Fishborne zoonotic trematodes (FZTs)* | Trematode | *Diverse* | *Diverse* | Intestinal trematode (fluke) disease | Yes | Fish |
| *Giardia duodenalis* | Protozoan | *Giardia* | *Hexamitidae* | Giardiasis | Yes | Diverse |
| *Hydatigera taeniaeformis* | Cestode | *Hydatigera* | *Taeniidae* | Diverse infections | No | Cats, dogs |
| *Leishmania donovani* | Protozoan | *Leishmania* | *-* | Leishmaniasis | Yes | Diverse |
| *Leishmania infantum* | Protozoan | *Leishmania* | *-* | Leishmaniasis | Yes | Canids |
| *Mesocestoides canislagopodis* | Cestode | *Mesocestoides* | *Mesocestoididae* | Intestinal infections | Yes (?) | Carnivore mammals, birds |
| *Myasis* | Ectoparasite | *-* | *-* | Myasis | Yes | Diverse |
| *Neospora caninum* | Protozoan | *Neospora* | *Sarcocystidae* | Neosporosis, Spontaneous abortion | No | Canids, livestock |
| *Schistosoma* | Cestode | *Schistosoma* | *Schistosomatidae* | Schistosomiasis (bilharzia) | Yes | Humans |
| *Toxoplasma gondii* | Protozoan | *Toxoplasma* | *Sarcocystidae* | Toxoplasmose | Yes | Cats |
| *Trichinella spiralis* | Nematode | *Trichinella* | *Trichinellidae* | Trichinellosis | Yes | Diverse |
| *Trichuris trichiura, Trichuris vulpis* | Nematode | *Trichuris* | *Trichuridae* | Trichuriasis | No | Dogs, humans |
| *Trypanosoma cruzi* | Eukaryotic | *Trypanosomen* | *-* | Chagas | Yes | Vertebrates |
| *Trypanosoma evansi* | Eukaryotic | *Trypanosomen* | *-* | Surra | Yes | Horses, camels |

**Table 4:** Full list of further relevant pathogen types found during the search process.

| **Pathogen** | **Type** | **Genus** | **Family** | **Disease** | **Zoonosis** | **Main host** |
| --- | --- | --- | --- | --- | --- | --- |
| *Trychophyton, Microsporum, Epidermophyton* | Fungi | Multiple | *Arthrodermataceae* | Dermatophytosis | Yes | Diverse |
| Prions | Prions | - | - | Transmissible spongiform encephalopathies (TSE); Prion disease | ? | Sheep, goats |

**Table 5:** Data pooling to report pathogen and parasite prevalences in main livestock and rodents/small mammals on farms found in publications worldwide during the literature review process. For each pathogen and animal type, the table lists the number (n) of field studies that identified prevalences, as well as the mean prevalences (%) and standard error (SE).

| **pathogen/parasite** | **cattle** | | | **pigs** | | | **poultry** | | | **goats** | | | **sheep** | | | **rodents/small mammals** | | |  |
| --- | --- | --- | --- | --- | --- | --- | --- | --- | --- | --- | --- | --- | --- | --- | --- | --- | --- | --- | --- |
|  |  |  |  |  |  |  |  |  |  |  |  |  |  |  |  |  |  |  |  |
|  | **n** | **mean %** | **SE** | **n** | **mean %** | **SE** | **n** | **mean %** | **SE** | **n** | **mean %** | **SE** | **n** | **mean %** | **SE** | **n** | **mean %** | **SE** |  |
| Akhmeta virus |  |  |  |  |  |  |  |  |  |  |  |  |  |  |  | 1 | 1.70 | - |  |
| *Anaplasma* spp. |  |  |  |  |  |  |  |  |  | 1 | 6.70 | - | 0 | - | - | 1 | 18.12 | - |  |
| Arthropodborne viruses | 1 | 4.90 | - |  |  |  |  |  |  |  |  |  |  |  |  |  |  |  |  |
| Avian influenza virus |  |  |  |  |  |  | 1 | 7.50 | - |  |  |  |  |  |  |  |  |  |  |
| *Bartonella* spp. |  |  |  |  |  |  |  |  |  |  |  |  |  |  |  | 4 | 34.44 | 12.93 |  |
| *Blastocystis* spp. | 1 | 25.00 | - |  |  |  |  |  |  |  |  |  |  |  |  | 1 | 37.90 | - |  |
| *Borrelia* spp. |  |  |  |  |  |  |  |  |  |  |  |  |  |  |  | 3 | 6.73 | 4.47 |  |
| *Brucella* spp. |  |  |  | 1 | 3.00 | - |  |  |  |  |  |  |  |  |  | 1 | 14.20 | - |  |
| *Campylobacter* spp. | 1 | 14.20 | - | 1 | 38.10 | - | 5 | 52.90 | 9.87 |  |  |  |  |  |  | 2 | 37.00 | 4.00 |  |
| *Clostridium* spp. |  |  |  | 2 | 51.85 | 7.23 |  |  |  |  |  |  |  |  |  | 3 | 31.00 | 4.95 |  |
| Coronavirus |  |  |  |  |  |  |  |  |  |  |  |  |  |  |  | 1 | 3.40 | - |  |
| *Coxiella* spp. | 2 | 8.55 | 3.45 |  |  |  | 1 | 25.00 | - |  |  |  |  |  |  | 1 | 9.32 | - |  |
| *Cryptosporidium* spp. | 2 | 6.90 | 3.30 |  |  |  |  |  |  |  |  |  |  |  |  | 5 | 16.50 | 5.83 |  |
| Dermatophytes |  |  |  | 1 | 18.60 | - | 2 | 43.15 | 38.45 |  |  |  |  |  |  | 1 | 37.40 | - |  |
| *Echinococcus* spp. |  |  |  |  |  |  |  |  |  |  |  |  |  |  |  | 1 | 0.07 | - |  |
| Ectoparasites |  |  |  | 1 | 54.80 | - |  |  |  |  |  |  |  |  |  | 4 | 31.25 | 13.38 |  |
| *Eimeria* spp. | 1 | 22.40 | - |  |  |  | 1 | 9.50 | - |  |  |  |  |  |  |  |  |  |  |
| Encephalomyocarditis virus |  |  |  | 3 | 54.17 | 12.16 |  |  |  |  |  |  |  |  |  |  |  |  |  |
| Enterobacteria |  |  |  | 1 | 67.00 | - |  |  |  |  |  |  |  |  |  | 1 | 51.00 | - |  |
| *Enterocytozoon* spp. | 1 | 11.90 | - |  |  |  |  |  |  |  |  |  |  |  |  | 5 | 17.56 | 5.81 |  |
| *Escherichia coli* |  |  |  |  |  |  |  |  |  |  |  |  |  |  |  |  |  |  |  |
| Flaviviruses, Alphaviruses |  |  |  |  |  |  |  |  |  | 1 | 1.20 | - |  |  |  | 1 | 2.20 | - |  |
| *Giardia* spp. |  |  |  |  |  |  |  |  |  |  |  |  |  |  |  | 1 | 10.80 | - |  |
| Helminths | 4 | 6.88 | 2.99 | 10 | 24.73 | 4.69 |  |  |  | 1 | 0.01 | - | 1 | 0.001 | - | 9 | 29.15 | 8.40 |  |
| Henipavirus |  |  |  |  |  |  |  |  |  |  |  |  |  |  |  | 1 | 4.10 | - |  |
| Hepatitis E virus |  |  |  | 1 | 34.50 | - |  |  |  |  |  |  |  |  |  | 4 | 14.58 | 4.99 |  |
| Influenza A virus |  |  |  | 1 | 75.70 | - |  |  |  |  |  |  |  |  |  | 1 | 11.04 | - |  |
| *Lawsonia* spp. |  |  |  | 1 | 90.74 | - |  |  |  |  |  |  |  |  |  | 3 | 63.96 | 10.44 |  |
| *Leishmania* spp. | 1 | 3.16 | - |  |  |  | 1 | 74.20 | - |  |  |  |  |  |  |  |  |  |  |
| *Leptospira* spp. | 19 | 23.78 | 4.49 | 7 | 45.75 | 9.08 |  |  |  | 6 | 19.34 | 9.29 | 4 | 21.66 | 13.87 | 18 | 24.12 | 4.34 |  |
| *Neospora caninum* | 6 | 21.13 | 7.74 | 2 | 13.80 | 5.14 |  |  |  |  |  |  | 1 | 15.22 | - | 3 | 5.80 | 3.60 |  |
| Pestivirus A/B | 1 | 7.70 | - |  |  |  |  |  |  |  |  |  |  |  |  |  |  |  |  |
| Porcine pseudorabies virus |  |  |  | 1 | 29.60 | - |  |  |  |  |  |  |  |  |  |  |  |  |  |
| *Rickettsia* spp./*Orientia* spp. |  |  |  |  |  |  |  |  |  |  |  |  |  |  |  | 1 | 16.94 | - |  |
| *Salmonella* spp. |  |  |  | 4 | 21.75 | 5.24 | 3 | 24.67 | 7.40 |  |  |  |  |  |  | 3 | 19.81 | 12.76 |  |
| Seoul virus |  |  |  |  |  |  |  |  |  |  |  |  |  |  |  | 1 | 19.00 | - |  |
| *Staphylococcus* spp. |  |  |  |  |  |  | 1 | 74.30 | - |  |  |  |  |  |  | 3 | 31.47 | 21.63 |  |
| *Toxoplasma* spp. | 4 | 23.58 | 8.69 | 19 | 20.82 | 5.53 | 2 | 22.64 | 10.94 | 3 | 54.34 | 17.58 | 2 | 63.21 | 1.25 | 13 | 12.52 | 4.42 |  |
| *Trypanosoma* spp. |  |  |  | 1 | 5.40 | - |  |  |  |  |  |  |  |  |  |  |  |  |  |
| Vaccinia virus |  |  |  |  |  |  |  |  |  |  |  |  |  |  |  | 1 | 5.20 | - |  |
| *Yersinia* spp. | 1 | 2.78 | - | 4 | 16.60 | 5.16 | 1 | 5.90 | - |  |  |  |  |  |  | 5 | 14.29 | 6.54 |  |
| Total n | 45 |  |  | 61 |  |  | 18 |  |  | 12 |  |  | 8 |  |  | 103 |  |  |  |
